# Pandemic (H1N1) 2009, Shanghai, China

**DOI:** 10.3201/eid1606.090991

**Published:** 2010-06

**Authors:** Yinzhong Shen, Hongzhou Lu

**Affiliations:** Shanghai Public Health Clinical Center, Shanghai, People’s Republic of China

**Keywords:** Pandemic (H1N1) 2009, influenza, human influenza, epidemiology, viruses, respiratory infections, China, dispatch

## Abstract

To understand the clinical and epidemiologic characteristics of pandemic (H1N1) 2009 virus infection, we retrospectively reviewed medical records of 237 patients with laboratory-confirmed cases reported in Shanghai, China, during May–July 2009. Surveillance activities effectively contained the outbreak and provided useful epidemiologic data for future strategies.

In early April 2009, human infections caused by pandemic (H1N1) 2009 virus were identified in the United States (*1*) and Mexico (*2*). The virus then spread rapidly around the world. The People’s Republic of China reported its first case of pandemic (H1N1) 2009 on May 10, 2009. As of July 31, a total of 2,090 confirmed cases had been reported in mainland China. Cases were detected in 25 provinces and municipalities; the largest numbers of cases were found in Guangdong Province, Beijing, and Shanghai. To understand the clinical and epidemiologic characteristics of infected patients, we reviewed medical records of 237 patients with laboratory-confirmed cases reported in Shanghai during May–July 2009.

## The Study

On April 30, 2009, guidelines for surveillance, reporting, diagnosis, and treatment of pandemic (H1N1) 2009 were published by the Ministry of Health of the People’s Republic of China (revised May 9) (*3*). On the basis of these guidelines, the Shanghai Bureau of Health issued a working document for prevention and control of pandemic (H1N1) 2009 in Shanghai.

Briefly, ill persons with a temperature >37.5°C were asked to visit fever clinics in local general hospitals. A suspected case of pandemic (H1N1) 2009 was defined as 1) an influenza-like illness (fever >37.5°C with >1 signs or symptoms, including sore throat, cough, runny nose, nasal congestion) in a person who had traveled to a country where >1 case had been confirmed in the past 7 days or 2) clinical symptoms or signs of influenza-like illness in a person epidemiologically linked to a patient with confirmed or suspected infection identified in the previous 7 days. A confirmed case was defined as laboratory confirmation of infection by PCR performed on a nasopharyngeal swab specimen at the Shanghai Center for Disease Control and Prevention (Shanghai CDC). All suspected cases were required to be reported to the Shanghai CDC within 24 hours after diagnosis.

Nasopharyngeal swabs obtained from patients with suspected cases were sent to the Shanghai CDC for detection of virus. Virus RNA was extracted and tested for all influenza types and specific subtypes by using a series of PCRs specific for matrix gene sequences of influenza A and B viruses. All patients with PCR results positive for pandemic (H1N1) 2009 virus were admitted to the Shanghai Public Health Clinical Center (SPHCC). We analyzed the clinical and epidemiologic features of confirmed cases reported in Shanghai during May 24–July 31, 2009.

In China, a national active surveillance system was established preemptively for recent travelers to areas affected by pandemic (H1N1) 2009. Thermal scanners were installed at all airports to detect fevers in travelers. Health questionnaires were administrated to travelers, and on the basis of answers to these questionnaires, all asymptomatic contacts of patients with suspected and confirmed pandemic (H1N1) 2009 were quarantined for 7 days. Symptomatic persons from an affected area were asked to visit fever clinics for confirmation of infection. Ambulances transported persons with suspected pandemic (H1N1) 2009 from airports to hospitals for screening. Health advisories encouraged travelers in whom influenza-like symptoms developed after arrival in Shanghai to seek medical care.

**Figure 1:**
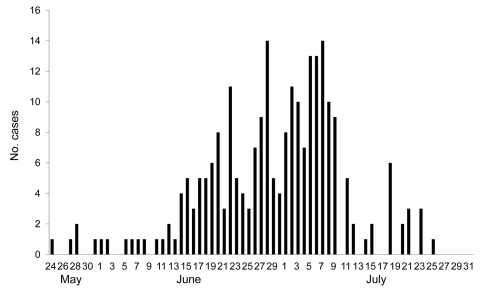
Daily number of laboratory-confirmed cases of pandemic (H1N1) 2009 virus infection, Shanghai, China, May 24–July 31, 2009.

Shanghai reported its first case of pandemic (H1N1) 2009 on May 24, 2009, in a traveler returning from Australia. As of July 31, 2009, SPHCC had identified 237 confirmed cases: 5 in May, 112 in June, and 120 in July (Figure 1). The maximum number of confirmed cases reported per date of onset (14) occurred on June 28 and July 7. Epidemiologic investigations suggested that the outbreak peaked in Shanghai in late June and early July.

**Figure 2:**
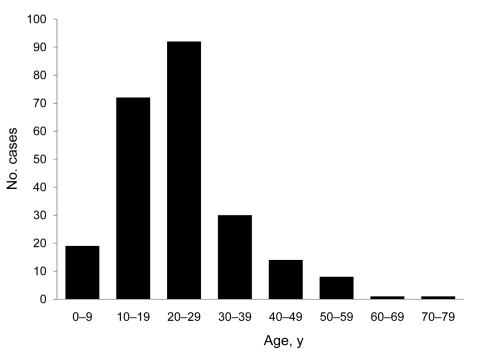
Number of laboratory-confirmed cases of pandemic (H1N1) 2009 virus infection, by age group, Shanghai, China, May 24−July 31, 2009.

Of the 237 case-patients, 129 (54.4%) were male. Median age of confirmed case-patients was 24 years (range 2–75 years). Eighty-two percent of case-patients were 10–39 years of age (Figure 2).

Patients were from 15 countries; 64.6% of patients were Chinese citizens, 10.1% were Australian citizens, 5.9% were American citizens, and 5.9% were Indonesian citizens. Among the 237 cases, 230 (97.0%) were identified as imported (i.e., confirmed case in a person with recent travel outside mainland China who had arrived in China during the surveillance period and had illness onset within 7 days after arrival). A total of 115 of these persons were from Australia; 36 from the United States; 18 from Canada; 11 from Indonesia; 10 from Singapore; 8 from the Philippines; 7 from the United Kingdom; 6 from Hong Kong; 4 each from Thailand, Taiwan, and South Korea; 2 each from Argentina and New Zealand; and 1 each from Japan, Mexico, and Italy. Seven (3.0%) patients (1 in June and 6 in July) contracted pandemic (H1N1) 2009 while in Shanghai and showed a clear epidemiologic link to a person with imported pandemic (H1N1) 2009. Of 230 imported cases, 124 (53.9%) were identified in airports upon arrival. No secondary cases occurred among hospital staff at SPHCC.

The most commonly reported symptoms were fever or history of fever (94.9%), dry cough (51.5%), sore throat (32.9%), runny nose (19.8%), and productive cough (18.1%) (Table). Only 2 case-patients (a 58-year-old man and a 49-year-old woman) had diarrhea (0.8%). One case-patient was a pregnant (13 weeks) Australian woman (31 years of age) in whom fever (>37.8°C) developed on the second day after she arrived from Sydney. Seventeen patients had underlying medical conditions: asthma (3 patients), obesity (3), allergic rhinitis (2), lymphoma (1), essential hypertension (5), hypothyroidism and hepatitis B (1), and gastric ulcer (2).

**Table:** Signs and symptoms in 237 patients with pandemic (H1N1) 2009 virus infection, Shanghai, China, May–July, 2009

Sign or symptom	No. (%)
>1 symptom	237 (100)
Fever or history of fever	225 (94.9)
Dry cough	122 (51.5)
Sore throat	78 (32.9)
Runny nose	47 (19.8)
Productive cough	43 (18.1)
Headache	14 (5.9)
Muscle pain	11 (4.6)
Altered consciousness	1 (0.4)
Diarrhea	2 (0.8)
Joint pain	2 (0.8)

Of the 237 case-patients, 236 (99.6%) received oseltamivir for 5 days; the pregnant woman refused antiviral therapy. Of 236 patients treated, 186 (78.5%) received oseltamivir within 48 hours after illness onset. Median time between onset of symptoms and start of oseltamivir treatment was 2 days (range 1–5 days). All patients had a mild illness. As of July 31, all patients had recovered and were discharged from the hospital. Mean ± SD length of hospital stay was 4.9 ± 1.7 days (range 3–9 days). No severe cases or deaths were reported. No patients were reported to have complications or to require intubation or oxygen.

## Conclusions

Our investigation indicates that the outbreak in Shanghai evolved similarly to outbreaks in other regions (*4**,**5*). The epidemiologic pattern in Shanghai did not differ from that in the Americas and Europe (*6**–**8*). Pandemic (H1N1) 2009 virus preferentially infects younger age groups. Most patients had mild symptoms and fully recovered within 1 week.

Clinical responses were favorable among these 237 case-patients, including 17 with underlying disease. Patients in this series seemed to benefit from early antiviral therapy. Early use of oseltamivir may have prevented complications.

Our study indicates that surveillance activities in Shanghai identified a substantial number of cases of pandemic (H1N1) 2009 among travelers early in the outbreak. The early response strategy in Shanghai has been containment; many case-patients were identified quickly after they arrived in China. Our data show that containment may have had a useful role in the initial phase of the outbreak. In Shanghai, patients with influenza were hospitalized for isolation purposes. Because the number of influenza cases has increased in Shanghai, the quarantine policy has been changed; since August 1, 2009, hospitalization for isolation purposes has not been mandated.

Cases of pandemic (H1N1) 2009 in Shanghai during May–July 2009 were identified rapidly and treated with oseltamivir, resulting in mild illness and absence of deaths. As the pandemic evolves, continued investigation is needed to describe its epidemiologic and clinical characteristics.
